# Clinical Characteristics of Patients with Vertebral Artery Hypoplasia

**DOI:** 10.3390/ijerph19159317

**Published:** 2022-07-29

**Authors:** Marcin Bakalarz, Jacek J. Rożniecki, Mariusz Stasiołek

**Affiliations:** 1 Department of Neurology, Teaching Hospital Nr. 1, Medical University of Lodz, Ul. Kopcińskiego 22, 90-153 Łódź, Poland; m.bakalarz1@gmail.com; 2 Department of Neurology, Stroke and Neurorehabilitation, Medical University of Lodz, Ul. Kopcińskiego 22, 90-153 Łódź, Poland; jacek.rozniecki@umed.lodz.pl; 3 Department of Neurology, Medical University of Lodz, Ul. Kopcińskiego 22, 90-153 Łódź, Poland

**Keywords:** ischemic stroke, cerebrovascular disease, vertebral artery hypoplasia

## Abstract

Vertebral artery hypoplasia (VAH) belongs to the relatively frequent Doppler ultrasonography (US) findings. However, its clinical significance remains controversial. This was a retrospective study analyzing clinical data of patients undergoing US because of cerebrovascular disease in a single academic neurology center. In the dataset of 2500 US examinations, 80 individuals with VAH (VA diameter < 2.0 mm) were identified (3.2% of all patients). Patients with significant vertebral artery asymmetry (SVAA, difference in VA diameters > 1.0 mm) (*n* = 80) and patients with normal VA diameter (*n* = 80) were also recruited. Clinical parameters including clinical signs and symptoms, concomitant diseases, imaging findings and the hospitalization outcome were compared between groups. The frequency of vertigo was highest in VAH group. Ischemic lesions of the cerebellum were found in 10% of VAH patients, 16% of SVAA patients and 5% of control subjects. Neurological deficits improved in over 60% of patients in each group, whereas ca. 30% of patients remained in a stable neurological status. The percentage of patients who deteriorated did not exceed 5% in any of the groups. The results of our study support a relatively high frequency of VAH. Our observations suggest coexistence of VAH with a higher frequency of neurological presentations associated with posterior arterial circulation of the central nervous system.

## 1. Introduction

Cerebrovascular disease (CVD) is considered to be one of the most common causes of hospitalization in neurological departments. Additionally, it accounts for a large percentage of visits in outpatient clinics. The human brain arterial supply is divided into anterior and posterior segments. Arteries of the brain and of a significant part of the spinal cord originate from two pairs of vessels-internal carotid and vertebral arteries. The posterior part of arterial system is formed by the pair of vertebral arteries (VA), which merge into one basilar artery. The vertebrobasilar system supplies 20% of blood volume entering the brain. The VAs supply blood to the brainstem and cerebellum, as well as a portion of the interbrain and cervical spinal cord segments, and additionally-fragments of the occipital and temporal lobes. [1,2].

**Figure 1:**
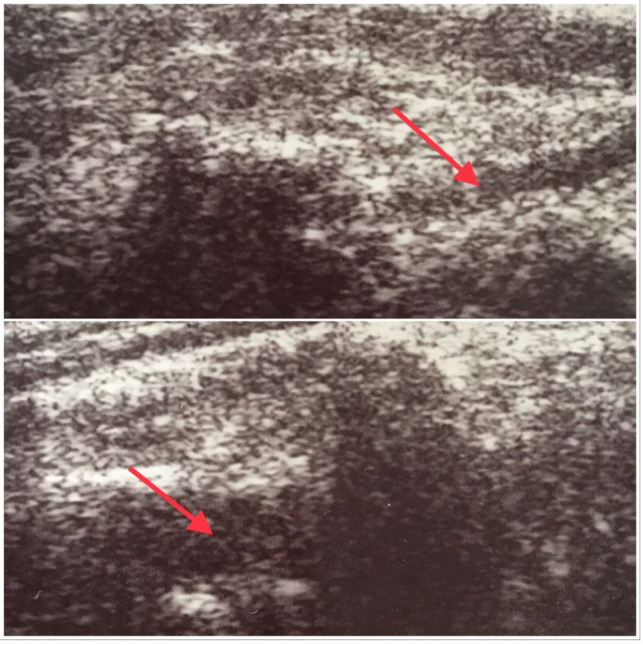
Comparison of the size of vertebral arteries: at the bottom-typical vertebral artery with the diameter 4.3 mm, at the top-hypoplastic vertebral artery with the diameter of 1.8 mm.

Ultrasound (US) examination of carotid and vertebral arteries with Doppler mode is considered as the elementary tool in the diagnostic workup of a patient with CVD. The most frequently observed abnormalities in carotid arteries in US examination are atherosclerotic lesions, which can lead to significant narrowing or sometimes complete obliteration of the vessel. Another group of abnormalities are congenital vascular anomalies. Among them, vertebral artery hypoplasia (VAH) is relatively frequent. Historically VAH, the anomaly described for the first time in the 19th century [3], has been the subject of much controversy for many years. Discussed issues include the definition and consistent criteria for the diagnosis of VAH. In the literature, the diameter of the hypoplastic VA is most often referred to as less than 2.0 mm (see Figure 1). However, other studies indicate that in some situations already a vessel diameter below 3.0 mm may significantly limit perfusion in the supplied area of the brain. Depending on the adopted criteria for diagnosis and the study population, the incidence of VAH was reported in a wide range of 1.9% to 26.5% of the general population [4,5,6,7,8,9,10,11].

Disturbances of the flow through the VA can lead to posterior inferior cerebellar artery (PICA) syndrome and basal artery syndrome. In the PICA syndrome, the most common symptoms and signs are vertigo, balance disturbances, vomiting. Ataxia may also occur. The severe cases may present as a full Wallenberg syndrome, characterized by Horner syndrome, paresis of the palate and posterior pharyngeal wall, trigeminal nerve dysfunction and hemiataxia ipsilaterally, as well as contralateral deficits of pain and temperature sensation on the trunk and limbs. Different clinical presentations may appear in the basilar artery syndrome, depending on the location of the occlusion in particular basilar artery segments or in its branches. Occlusion of the main basal artery trunk is the cause of one of the most severe types of ischemic stroke, with very high mortality [1,2,12,13].

The results of studies published so far suggest a possible relationship between VAH and increased risk of ischemic stroke or transient ischemic attack (TIA) in posterior vascular area of the brain [4,5,6,7,8,9,10,12,13,14,15,16,17,18]. An existence of a similar relationship was also suggested in the case of significant asymmetry of the VAs (diameter ratio higher than 1:1.7) [4,6,7,9,19]. Taking into consideration the previously reported relatively high incidence of VAH and unresolved controversy regarding its clinical relevance, in our study we characterized the clinical picture of patients with VAH in relation to individuals with significant vertebral artery asymmetry (SVAA) and a group of patients without VA abnormalities.

## 2. Materials and Methods

### 2.1. Patients and Data Collection

This was a single center retrospective study. The Bioethics committee of the Medical University of Lodz approved all the procedures included in the protocol of the study (Approval no. RNN/351/18/KE). The participants (*n* = 240) were recruited among the patients hospitalized in the Department of Neurology, Teaching Hospital No. 1 of the Medical University of Lodz, Poland between November 2013 and December 2019. All the patients underwent US examination of carotid arteries and VAs as routine medical procedure associated with differential diagnostics of CVD. The participants of the study were assigned into three groups based on the US findings regarding VAs morphology: (1) VAH, (2) SVAA (3) control group with normal VA morphology. For the purposes of the study following definitions were used: a diameter of VA smaller than 2.0 mm was considered as VAH. A difference in VA diameters greater than 1.0 mm was considered as SVAA. Such definition of SVAA was adopted because of the practical nature of this study. For an experienced US examiner, the threshold of at least 1.0 mm enables easy and repeatable detection and proper documentation of diameter difference between VAs. The control group included patients with both VAs of normal diameter and without the significant (i.e., ≥1.0 mm) difference in diameter. In the data set of 2500 US examinations performed in the indicated period, 80 cases of VAH were identified. Importantly, the study did not include examinations where at least one VA was not visualized (*n* = 85; 3.4% of the whole data set). In the same data set, the SVAA group and the control groups were recruited consecutively in a random way until each group reached the *n* = 80. Demographic (age, gender) and clinical parameters (neurological signs and symptoms, cerebral imaging findings, comorbidities) were included into analysis. Results of cerebral imaging were available in all the participants: magnetic resonance imaging (MRI; 1.5 T) in 101 patients (39 patients of VAH group, 34 patients of SVAA group and 28 patients of control group) and cerebral computed tomography (CT) in the rest of the participants. Hospitalization outcome was assesses based on a dynamic of neurological status measured with the National Institutes of Health Stroke Scale (NIHSS).

### 2.2. US Methodology

All the US examinations were performed by the same certified US specialist with the use of the same cardiovascular ultrasound system Siemens CV70 equipped with a linear probe suitable for vascular diagnostics (model L10-5 Linear Array Probe 5–10 Mhz). The ultrasound equipment was adjusted with the manufacturer protocol dedicated to carotid arteries evaluation. The US examination was performed in patients lying down. The angle of insonation was 60 degrees.

### 2.3. Statistical Analysis

Statistical analyses were performed using R 3.5.2. statistical software (R Core Team (2018). R: Language and environment for statistical computing by R Foundation for Statistical Computing, Vienna, Austria). The normality of distributions in the analyzed patient groups for quantitative variables (age) had been checked using the Shapiro-Wilk test, based on which it was found that age had no normal distribution. Nominal data were presented as *n* (% of group). Quantitative data were described using basic descriptive statistics: arithmetic mean, standard deviation (SD), median, quartiles (first and third-Q1, Q3) and minimum and maximum values. In order to identify statistically significant differences between the 3 groups of patients, chi-square tests or Fisher’s exact tests for nominal variables, and Kruskall-Wallis test for quantitative variables were performed for individual variables. In the case of a statistically significant difference for a given variable, a series of post-hoc tests were performed for individual pairs of study groups. All statistical tests performed were two-sided. Results with a significance level (*p*) < 0.05 were considered significant.

## 3. Results

### 3.1. VAH Prevalence and Demographic Factors

In the data set of 2500 US examinations performed between November 2013 and December 2019, 80 patients with VAH were identified, which constituted 3.2% of all the examined patients. The SVAA group and control group were recruited from the same data base. Over half of the patients in each group were women. Female patients represented 58% (*n* = 46) of the VAH group, 54% (*n* = 43) of the SVAA group and 59% of the control group (*n* = 47) (Table 1). No statistically significant difference in the gender structure among the three study groups was found (*p* = 0.802). Also the average age of patients in the study groups did not differ significantly (*p* = 0.352). The median age of patients was 65 years for the VAH group, with the youngest patient in this group being 27 years old and the oldest being 92 years old. In the SVAA group, the patients’ age had a median of 67 years and ranged from 24 to 94 years. The control group consisted of patients with a median age of 67 years and range from 23 to 89 years old (Table 2).

VAH was slightly more common in the right VA than in the left one (53% and 48%, respectively). In the case of SVAA the larger diameter of the left VA was revealed more frequently (65% vs. 35%). There were no differences in the incidence of hemodynamically significant internal carotid artery stenosis between the study groups (*p* = 0.628). Significant stenosis affected 14% of patients in the VAH group (*n* = 11), 19% of patients in the SVAA group (*n* = 15), and 18% of patients in the control group (*n* = 14).

### 3.2. Primary Diagnoses

The analysis of the primary diagnoses revealed that the most common cause of hospitalization in each group was ischemic stroke, which affected half of the patients in the SVAA group (51%, *n* = 41), 41% of patients in the VAH group (*n* = 33) and 49% in the control group (*n* = 39). The second most common primary diagnosis was TIA, affecting 19–20% of patients, depending on the group. The frequency of other diagnoses did not reach more than 10% of patients in none of the groups. The most common other diagnoses in particular groups were as follows: in the VAH group cranial nerve disorders (8.8%, *n* = 7), epilepsy (5.0%, *n* = 4) and demyelinating disease (5.0%, *n* = 4); in the SVAA group epilepsy (7.5%, *n* = 6), hemorrhagic stroke (6.3%, *n* = 5) and cranial nerve disorders (3.8%, *n* = 3); in the control group cranial nerve disorders (5.0%, *n* = 4) and epilepsy (5.0%, *n* = 4). There were no statistically significant differences in the frequency of specific primary diagnoses among the study groups.

### 3.3. Neurological Symptoms

In the further analysis, the frequency of particular neurological signs and symptoms was compared in the study groups. The neurological presentations were grouped into broader categories: limb paresis, cranial nerves disorder, consciousness disturbances, aphasia, sensation disorders, vertigo, headache, visual field disorders, ataxia, and monocular vision. In all groups, “limbs paresis” was the most frequent category of neurological deficits and it affected 48% of patients in the VAH group (*n* = 38), 55% in the SVAA group (*n* = 44), and 50% of patients in the control group (*n* = 40). The second most common neurological presentation in all groups was “disorder of cranial nerves”, described in 44% (*n* = 35) of the patients in the VAH group, 36% (*n* = 29) in the SVAA group and 40% (*n* = 32) in the control group. There was no statistically significant difference in the frequency of paresis nor cranial nerves disorder among the study groups. Disturbances of consciousness, aphasia, sensory disturbances or vertigo were less common and affected 11% to 28% of patients in particular study groups. Only the frequency of vertigo differed significantly among the three groups of patients (*p* = 0.025) (Table 3). The post-hoc analysis demonstrated that the frequency of vertigo was significantly higher in VAH group (28%, *n* = 22) than in the SVAA group (11%, *n* = 9). However the frequency of vertigo was not significantly different in control group when compared neither with VAH nor SVAA group. Additionally, the comparison of cumulative data from both “abnormal VA” groups (VAH + SVAA) with controls did not reveal significant differences in vertigo frequency (data not shown). Other neurological signs and symptoms (headache, visual field disturbances, ataxia, and monocular vision) affected no more than 10% of patients in each group and their frequency did not differ among the groups (Table 3).

### 3.4. Comorbidities

Comorbidity spectrum was analyzed in the study groups with the focus laid on: hypertension, diabetes, atrial fibrillation, atherosclerosis and heart failure. Hypertension and atherosclerosis were the most common concomitant conditions in all the groups. Atherosclerosis was present in 45–58% of patients, and its frequency did not differ significantly among study groups (*p* = 0.247). Whereas, significant difference was found in the frequency of hypertension (*p* = 0.040). The post-hoc analysis showed that hypertension was significantly more frequent in the control group (*n* = 59), than in the SVAA group (55%, *n* = 44), *p* = 0.021. There were no other significant differences regarding this parameter. Diabetes, atrial fibrillation and heart failure were less common and affected no more than 25% of patients in each group. There were no significant differences in the frequency of these conditions among the study groups (Table 4).

### 3.5. Brain Ischemic Lesions

The assessed parameters encompassed additionally the main findings of cerebral MRI and/or CT examinations in the study groups. Supratentorial lesions suggestive of ischemic changes were the most frequently reported findings. This kind of imaging pathology was described in 68% of patients in the VAH group (*n* = 54), 78% of patients in the SVAA group (*n* = 62) and 70% of patients in the control group (*n* = 56). Ischemic brainstem lesions were visualized in a very small percentage of patients (1–4% depending on the group). However, ischemic lesions of the cerebellum were found in 10% of patients in the VAH group (*n* = 8), 16% of patients in the SVAA group (*n* = 13) and 5% (*n* = 4) of subjects in control group. Although there were no statistically significant differences in the frequency of these lesions a trend towards increased percentage of cerebellar lesions was observed in patients in VAH and SVAA group as compared to control group (the *p* value 0.075) (Table 5). However, the comparison of cumulative imaging data of VAH + SVAA groups and control group did not reveal significant differences in frequency of cerebellar lesions (data not shown).

### 3.6. Clinical Outcomes

The clinical outcome of hospitalization was also included in the analysis. There were no statistically significant differences of neurological status assessed with NIHSS in the outcome of hospitalization among the study groups. The neurological deficits improved in over 60% of patients in each group, whereas ca. 30% of patients remained in a stable neurological status. The percentage of patients who deteriorated did not exceed 5% in any of the groups (Table 6).

## 4. Discussion

In our study, we retrospectively analyzed a large database containing clinical data obtained from 2500 patients who underwent US examination of carotid and vertebral arteries. In this dataset, 80 cases of VAH were identified which accounted for 3.2% of patients. It is possible that the real percentage of VAH was slightly higher since in our analysis we excluded cases in which at least one VA was not visualized because of difficult anatomical conditions. In this situation, the hypoplastic artery may be difficult to detect by US examination. Such cases encompassed 85 examinations i.e., 3.4% of all patients in the database. Still, our results fit into the VAH frequency range known from previous reports: 1.9% to 26.5% of cases [4,5,6,7,8,9,10,11]. One of the main reasons of the considerable differences in the reported VAH frequency is most probably the lack of established diagnostic criteria. Although in the majority of cases VAH has been defined as artery with diameter below 2.0 mm, some studies used other thresholds e.g., 2.5 mm [20]. Other possible explanation for such differences in VAH frequency is the diversified examination methodology. More advanced diagnostic techniques such as angio-MRI or angio-CT improve the sensitivity of VAH detection in comparison to US, particularly in the case of difficult anatomical conditions [21]. As an example, in one of the studies based on angio-CT, the VAH frequency was calculated as 20.9% in a group of patients with vertigo and 7.5% in a control group (diameters < 2 mm were accepted as hypoplastic) [13]. In another study, application of MRI TOF examination resulted in the detection of VAH (defined again as diameter < 2mm) in 26.5% healthy subjects. Most importantly, in the same study VAH frequency in patients with posterior circulation stroke reached 45.6%, whereas in patients with anterior circulation stroke, VAH frequency was similar to the control group (27.1%) [8]. Such potentially clinically relevant observations were reported also in other studies [18,22]. Interestingly, the results of our study confirmed earlier observations that the VA usually has a larger diameter on the left side and consequently VAH is more common on the right side. However, the difference was less pronounced than in earlier studies, which demonstrated that right VAH may by even two times more frequent than the left one [4,22]. In the VAH group the highest percentage of patients with vertigo was observed, although the difference was statistically significant in comparison with SVAA but not control group. Nonetheless, this observation stays in concordance with clinical associations reported in previous studies [13]. The incidence of other neurological symptoms i.e., paresis, cranial nerves disorders, disturbances of consciousness, aphasia, sensation disturbances, headache, visual field disturbances, ataxia and monocular vision were similar in all three patient groups of our study. We observed a trend towards a more frequent presence of cerebellar ischemic lesions in brain imaging among patients with VAH and SVAA, although the difference did not reach the statistical significance. On the other hand, in contrast to some of the earlier studies, in our population we did not observe differences in the prevalence of brain stem ischemic lesions between patients with or without VAH [10]. However it is crucial to indicate that a significant part of the patients examined in our study did not have MRI of the head. Thus, minor ischemic lesions, not visible on CT scans, could not be detected in this subgroup of patients. This aspect seems to be particularly worth of further investigation in the light of the recently published study suggesting VAH as a predisposing factor for atherosclerotic stenosis in the posterior circulation [18]. Moreover, in another recent US analysis, abnormal function of dominant VA in VAH patients was indicated, which may add to the risk of vertebrobasilar insufficiency in this group [23]. The comparison of concomitant disorders in the study groups showed that arterial hypertension was more frequent in the control group than in SVAA group. In another study patients with VAH were significantly younger (mean age 60 ± 13 vs. 64 ± 14 years,), more often smokers (31 vs. 53%) and less often hypertensive (77 vs. 41%) [22]. These observation may stay in line with our findings regarding higher frequency of hypertension in patients with normal VAs. The reason for this phenomenon is not clear, but we can speculate that hypertension predisposes patients with normal VAs to clinical situations demanding US examination.

Although the database used in the study encompasses a relatively large number of US examinations performed by the same examiner with the same US equipment, there are several limitations which have to be considered in the interpretation of the results. The study was based on the retrospective analysis of the medical data obtained in a single academic center located in an industrial area. Thus it is impossible to extrapolate the findings of the study on the general population. Additionally, the range of retrospective clinical data available for the analysis was limited, so many potential factors influencing the occurrence and clinical course of cerebrovascular disease were not included in the analysis. Due to the retrospective nature of the study, no intra-observer reliability analysis could be performed. Additionally, as already mentioned above, US examination by itself is associated with technological limitations as compared with more advanced imaging methods as for example angio-CT or angio-MRI. However, we believe that the presented results considerably extend the knowledge of VAH, which significance in the everyday medical practice remains insufficiently understood.

## 5. Conclusions

The results of our study support a relatively high frequency of VAH among patients hospitalized for differential diagnostics of CVD. Additionally, our observations suggest coexistence of VAH with a higher frequency of neurological presentations associated with posterior arterial circulation of brain, especially vertigo. The frequency of comorbidities such as hypertension, atherosclerosis, diabetes, heart failure, and atrial fibrillation was similar in our population of VAH patients and non-VAH individuals, which stays partially in contrast to previous reports. These observations deserve to be further investigated in more detailed analyses performed on bigger populations of patients, especially with cerebrovascular diseases.

## Figures and Tables

**Table 1:** Gender structure.

Gender	VAH		SVAA		Control		*p*
	*n*	%	*n*	%	*n*	%	
Female	46	57.5%	43	53.8%	47	58.8%	0.802
Male	34	42.5%	37	46.3%	33	41.3%	

**Table 2:** Median age of patients.

	VAH	SVAA (Q1, Q3)	Control (Q1, Q3)	*p*
Age (Q1–Q3)	65.0 (52.8–72.0)	67.0 (57.0–72.3)	67.0 (58.0–79.0)	0.352

**Table 3:** Frequency of neurological symptoms.

Neurological Symptoms	VAH		SVAA		Control Group		*p*
	*n*	%	*n*	%	*n*	%	
Limb paresis	38	47.5%	44	55.0%	40	50.0%	0.627
Cranial nerves disorder	35	43.8%	29	36.3%	32	40.0%	0.532
Consciousness disturbances	14	17.5%	11	13.8%	21	26.3%	0.120
Aphasia	19	23.8%	17	21.3%	18	22.5%	0.931
Sensation disorders	11	13.8%	15	18.8%	14	17.5%	0.677
Vertigo	22	27.5%	9	11.3%	13	16.3%	0.025
Headache	6	7.5%	6	7.5%	5	6.3%	0.939
Visual field disturbances	5	6.3%	2	2.5%	3	3.8%	0.614
Ataxia	3	3.8%	8	10.0%	2	2.5%	0.156
Monocular vision	3	3.8%	2	2.5%	0	0.0%	0.376

**Table 4:** Frequency of comorbidities.

	VAH		SVAA		Control		*p*
	*n*	%	*n*	%	*n*	%	
Hypertension	54	67.5%	44	55.0%	59	73.8%	0.040
Artherosclerosis	38	47.5%	46	57.5%	36	45.0%	0.247
Diabetes	18	22.5%	14	17.5%	20	25.0%	0.503
Atrial fibrillation	14	17.5%	6	7.5%	14	17.5%	0.112
Heart failure	11	13.8%	12	15.0%	9	11.3%	0.777

**Table 5:** Cerebral imaging findings.

Type o Brain Lesions	VAH		SVAA		Control Group		*p*
	*n*	%	*n*	%	*n*	%	
Supratentorial ischemic lesions	54	67.5%	62	77.5%	56	70.0%	0.344
Brainstem ischemic lesions	3	3.8%	3	3.8%	1	1.3%	0.555
Cerebellum ischemic lesions	8	10.0%	13	16.3%	4	5.0%	0.075

**Table 6:** Outcome of hospitalization.

Outcome of Hospitalization	VAH		SVAA		Control		*p*
	*n*	%	*n*	%	*n*	%	
Deterioration	4	5.0%	3	3.8%	4	5.0%	0.981
Recovery	52	65.0%	51	63.8%	53	66.3%	
Stable	24	30.0%	26	32.5%	23	28.8%	

## Data Availability

Data are stored in the Department of Neurology, Medical University of Lodz.
